# Evidence that Chemical Chaperone 4-Phenylbutyric Acid Binds to Human Serum Albumin at Fatty Acid Binding Sites

**DOI:** 10.1371/journal.pone.0133012

**Published:** 2015-07-16

**Authors:** Debasish Roy, Vinod Kumar, Joel James, Mohamed Sham Shihabudeen, Shweta Kulshrestha, Varun Goel, Kavitha Thirumurugan

**Affiliations:** 206, Structural Biology Lab, Center for Biomedical Research, Division of Biomedical Sciences, School of Bio Sciences and Technology, VIT University, Vellore-632014, Tamil Nadu, India; Islamic Azad University-Mashhad Branch, Mashhad, ISLAMIC REPUBLIC OF IRAN

## Abstract

Endoplasmic reticulum stress elicits unfolded protein response to counteract the accumulating unfolded protein load inside a cell. The chemical chaperone, 4-Phenylbutyric acid (4-PBA) is a FDA approved drug that alleviates endoplasmic reticulum stress by assisting protein folding. It is found efficacious to augment pathological conditions like type 2 diabetes, obesity and neurodegeneration. This study explores the binding nature of 4-PBA with human serum albumin (HSA) through spectroscopic and molecular dynamics approaches, and the results show that 4-PBA has high binding specificity to Sudlow Site II (Fatty acid binding site 3, subdomain IIIA). Ligand displacement studies, RMSD stabilization profiles and MM-PBSA binding free energy calculation confirm the same. The binding constant as calculated from fluorescence spectroscopic studies was found to be k_PBA_ = 2.69 x 10^5^ M^-1^. Like long chain fatty acids, 4-PBA induces conformational changes on HSA as shown by circular dichroism, and it elicits stable binding at Sudlow Site II (fatty acid binding site 3) by forming strong hydrogen bonding and a salt bridge between domain II and III of HSA. This minimizes the fluctuation of HSA backbone as shown by limited conformational space occupancy in the principal component analysis. The overall hydrophobicity of W214 pocket (located at subdomain IIA), increases upon occupancy of 4-PBA at any FA site. Descriptors of this pocket formed by residues from other subdomains largely play a role in compensating the dynamic movement of W214.

## Introduction

**Fig 1:**
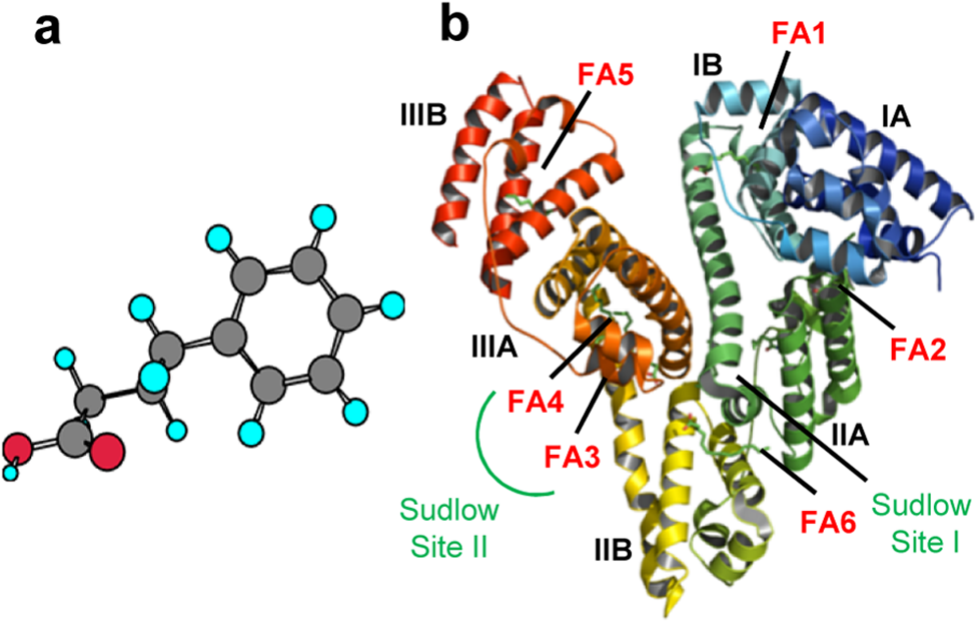
A) Ball and Stick representation of 4PBA; Carbon = Grey, Hydrogen = Cyan, Oxygen = Red b) Cartoon model of HSA-MYR complex (PDB: 2BXP) showing different subdomains and major fatty acid binding sites.

Endoplasmic reticulum (ER) is the chief cellular organelle involved in the biosynthesis of polypeptides and lipids. After protein translation by ribosomal machinery on cytosolic surface of the ER, the unfolded polypeptide gets translocated in the ER lumen for folding, assisted by molecular chaperones and foldases. The ER maintains a rigid quality control and proteins that are well-folded only exits the ER for various cellular fates. This strict homeostasis is disrupted in various disease states like diabetes, obesity, cancer, neurodegenerative disorders etc [1–3], where there is an accumulation of unfolded proteins owing to the availability of excess metabolites or due to an altered redox status. To counteract, the cell triggers an adaptive response known as the Unfolded Protein Response (UPR). UPR augments the ER to cope with the misfolded proteins by increasing the amount of chaperones, decreasing the entry of new proteins or by degrading irreversibly misfolded proteins in the ER lumen. Recently, several small molecules have been identified that act as chaperones. They have potential to reduce UPR and promote protein folding by reducing the energy barrier of the intermittent transition states that occur as the proteins fold into their native conformation. Several studies have reported that 4-Phenylbutyric acid (PBA) (Fig 1A), a FDA approved drug for treating urea cycle disorder [4, 5] has efficacy in assisting protein-folding in ER, thereby decreasing UPR levels both *in vitro* and *in vivo*.

Drug bioavailability is majorly affected by its binding to Human serum albumin (HSA)[6]; the most abundant protein in plasma. Apart from acting as a drug transporter, HSA transports a large variety of endogenous and exogenous ligands like fatty acids, metabolites and hormones [7–9] to target organs. HSA has three homologous domains (I, II and III) and each domain is divided into two subdomains (A and B) (Fig 1B). Drug binding to HSA takes place mostly at subdomain IIA and IIIA which are commonly referred to as Sudlow site I and II respectively [9]. Molecular interactions at these sites are monitored using fluorescence properties of intrinsic fluorescence probe, W214 (located in subdomain IIA). Some drug molecules which are known subdomain IIIA binders can bind to subdomain IB to make it as a third major drug site [10]. Apart from drug binding, HSA also acts as a carrier for a number of physiological ligands like heme, bilirubin, fatty acids etc [11]. Primary binding sites for fatty acids are located in subdomain IA and IIIA of HSA. This versatile binding behaviour of HSA plays a major role in determining the rate of delivery of ligands and hence has been extensively studied for its binding with various ligands over the last three decades.

The binding nature of 4-PBA to HSA is still not known. Due to its promise in many preclinical studies of diseases in which ER stress is a central mechanism of manifestation, 4-PBA binding to HSA can provide important clues on its pharmacokinetics. With this view, binding studies of 4-PBA with HSA is performed.

## Materials and Methods

### Materials

Human serum albumin (fatty acid free), 4-Phenylbutyric Acid (4-PBA) and Palmitic acid were purchased from Sigma Aldrich, India. Quercetin was purchased from SRL, India. For HSA, 200μM stock solutions were prepared in Tris HCl (0.05 molL^-1^) and NaCl (0.1 molL^-1^), pH 7.4 and stored at -20°C in aliquots. The same buffer was used for UV-Vis and Fluorescence assays. For CD analysis, Tris HCl (0.01 molL^-1^) and NaCl (0.1 molL^-1^), pH 7.4 was used. Stock solutions of PBA (2mM), Quercetin (2mM) and Palmitic acid (2mM) were prepared in absolute ethanol and stored at room temperature in experimental aliquots.

### Methods

#### UV-Vis absorbance spectrophotometry

All UV-Vis spectroscopic measurements were carried out using Thermo scientific Evolution 260 Bio double beam spectrophotometer (USA) with a 1 cm path length. Binding experiments were performed using a fixed concentration of HSA (1 μM) and 4-PBA in the range 1–20 μM. The UV-Vis spectral scan was recorded from 500–200 nm at 25 ± 0.2°C.

#### Fluorescence assays

Fluorescence measurements were carried out in a JASCO spectrofluorimeter FP 8600 (Tokyo, Japan), using 1 cm path length quartz cuvette. The emission spectrum was studied with a fixed volume of HSA (1 μM) in the presence of increasing concentrations of Quercetin (1–20 μM), 4-PBA (1–20 μM) and Palmitic acid (1–20 μM). For titrations, ligands were successively added (5μl) to obtain the given concentration range from ethanol stock. After every addition the solutions were thoroughly mixed and incubated for 5 minutes at 25 ± 0.2°C before collecting spectra. Fluorescence spectra were recorded with an excitation wavelength of 295 nm and an emission range of 300–500 nm. For fixed wavelength measurements, emission wavelengths of 345 nm and 330 nm were used. The effect of ethanol on the emission spectra was evaluated and it was found to be negligible.

#### Ligand Displacement Studies

Two separate ligand displacement assays were conducted: (1) by adding varying concentrations of 4-PBA (2–20 μM) to a mixture of HSA (1 μM) + Quercetin (20 μM); (2) by adding varying concentrations of Palmitic acid & 4-PBA (1–8 μM) to HSA (1 μM) + Dansylglycine (1 μM) complex. HSA + ligand complexes were incubated at 25°C for 30 mins prior data collection. The stability of the complex was evaluated by measuring fluorescence emission spectra with time (for 1 min) to ensure that it reached equilibrium before titration. Percentage initial fluorescence was calculated by: [Fluorescence in the presence of added ligand] / [Fluorescence in the absence of added ligand] * 100 [12].

#### Circular Dichroism

CD spectra of HSA and ligand complexes were recorded with a JASCO J-715 spectropolarimeter (Tokyo, Japan). For measurements in the far-UV region (178–280 nm), a quartz cell with a path length of 0.1 cm was kept in constant nitrogen flow atmosphere. HSA and 4-PBA was mixed in an equal stoichiometry. Accumulation of the scan with the scan speed of 50 nm per min was performed and the data was collected from the 260–200 nm spectral range. All the CD measurements were carried out at 25 ± 0.2°C.

#### Molecular Dynamics Simulation

MD simulation was carried out using GROMACS 4.5.6 [13] to understand the dynamic behaviour of HSA in a 4-PBA bound state. Amber 99sb force field [14] parameters were used for protein structure and GAFF parameters [15] were used for small molecules using ACPYPE tool [16]. The 4-PBA-HSA complexes were solvated in a cubic box with SPCE water model under periodic boundary conditions. The system was neutralized by adding 14 sodium ions. The neutralized protein–ligand complexes were energy minimized by using steepest descent method. After energy minimization, the systems were equilibrated by Position restrained molecular dynamics at constant temperature of 300 K and a constant pressure of 1 atm for about 100 ps. Finally, the equilibrated systems were subjected to MD for 7,000 ps using NPT ensemble.

#### Principal Component Analysis (PCA)

MD simulation trajectory file was used to identify the motion of unliganded HSA and 4-PBA-HSA complex (4-PBA bound to different FA sites). PCA analysis in protein dynamics (analyzed using Gromacs tools: g_covar and g_anaeig) is used to identify possible collective motions of residues of a protein due to certain changes inflicted upon ligand binding [17]. A co-variance matrix for protein backbone was obtained, transformed in Cartesian co-ordinate space (before and after simulation) and diagonalized. The direction of motion was characterised by representing the vectors of every single components of motion. The eigenvalues represent the amplitude of fluctuation along their corresponding eigenvectors. Therefore, the contribution to the internal motion of HSA upon complexation is traced by PCA. The major combined movements arise along the directions of eigenvectors associated to the largest eigenvalue. The first 10 eigenvectors were used to represent the protein motion.

#### MM-PBSA binding free energy calculation

Binding free energy at each ligand binding site was computed by using GMXPBSA 2.1 [18] with trajectory files obtained after simulation. Polar solvation energy values was calculated using the implicit solvation Poisson–Boltzmann model (PB) and nonpolar solvation energy value was calculated based on the solvent accessible surface area (SASA); finally all these were combined to obtain final binding free energy value.

Binding free energy of 4-PBA at each fatty acid binding site was calculated by using [19]:

$${\Delta \text{G}}_{\text{bind}}={\text{G}}^{\text{complex}}-{\text{G}}^{\text{HSA}}-{\text{G}}^{4\text{PBA}}$$ (Eq 1)

G represents free energy of each state, which was computed by:

$$\text{G}={\text{E}}_{\text{MM}}+{\text{G}}_{\text{PB}}+{\text{G}}_{\text{SA}}-\text{TS}$$ (Eq 2)

$${\text{E}}_{\text{MM}}={\text{E}}_{\text{vdw}}+{\text{E}}_{\text{ele}}+{\text{E}}_{\text{int}}$$ (Eq 3)

E_MM_ is the molecular mechanical energy, G_PB_ and G_SA_ are the polar and nonpolar terms of the free energy and TS is the entropic contribution of solute. E_MM_ was obtained by adding contributions from internal energies including bond, angle, and torsional angle energies (E_int_), electrostatic energy (E_ele_), and van der Waals energy (E_vdw_).

## Results

### Interaction between 4-Phenylbutyric acid and HSA

#### UV-Vis spectroscopy

**Fig 2:**
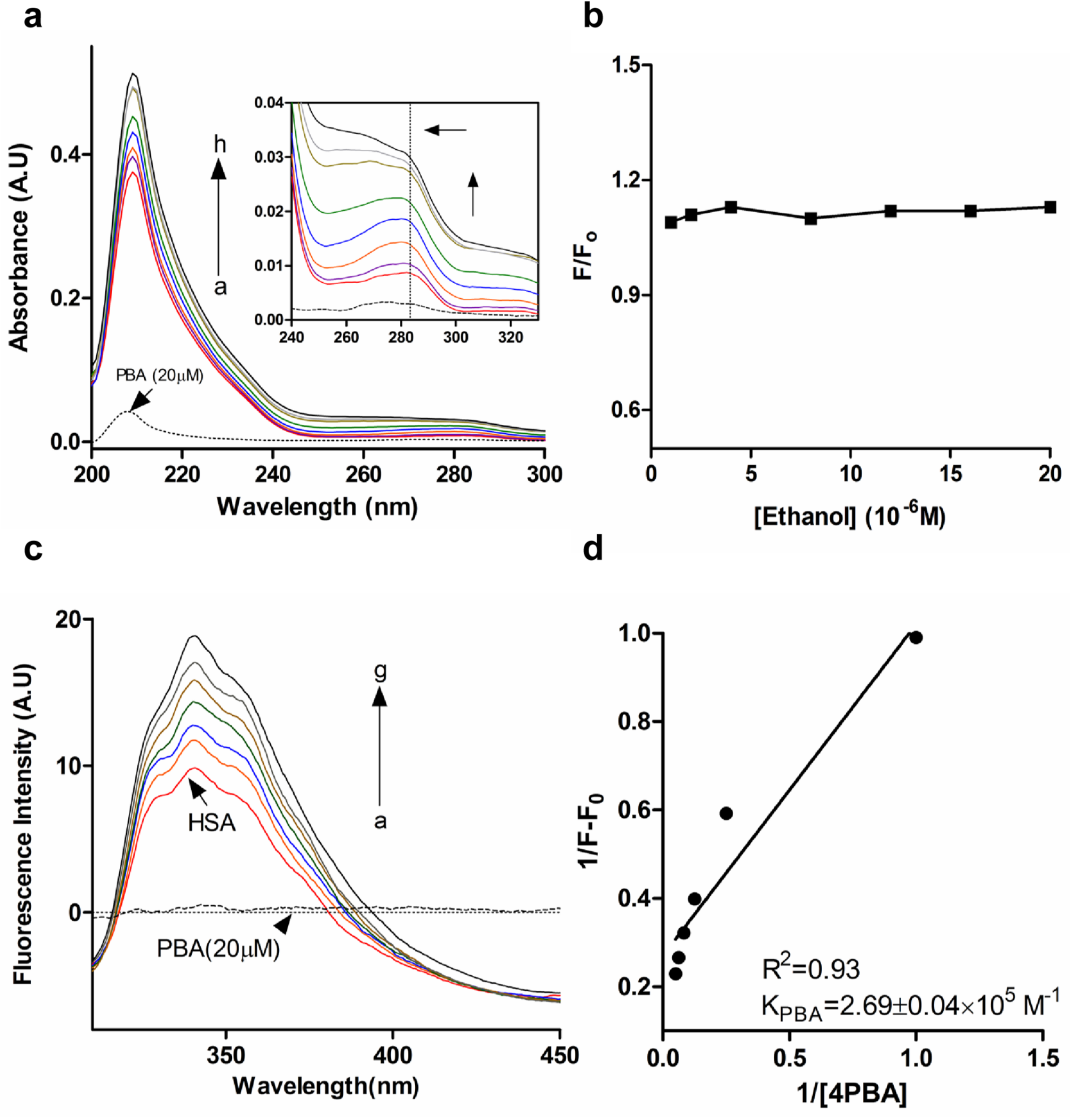
Interaction of 4PBA with HSA. a) UV-Vis spectra of HSA in presence of 4PBA. Cuvette concentration, cHSA: 1μM (a) and c4PBA (1,2,4,8,12,16,20 μM): b→h; pH 7.4 at 25°C. Inset shows slight blue shift at the Tryptophan absorption region indicated by the arrow. b) Effect of ethanol on the fluorescence emission intensity of HSA. cHSA = 1μM, cEthanol =, 1,2,4,8,12,16,20 μM (pH 7.4, 25°C) c) Fluorescence emission spectra of HSA in presence of 4PBA. cHSA: 1μM (a) and c4PBA (2,4,8,12,16,20 μM): b→g; pH 7.4 at 25°C.Dotted line represents contribution of4PBA (20μM) at the emission range of HSA. d) Plot of 1/F-F_0_ vs 1/ [4PBA]

UV-Vis and Fluorescence spectroscopy is used to understand structural changes induced to a protein upon ligand binding and complex formation. Aromatic residues like tryptophan are very sensitive to slight changes in the polar microenvironment. This rearrangement in the polar microenvironment of tryptophan is attributed to changes in the protein secondary structure caused by ligand binding. Absorption spectrum of HSA is obtained by titrating with increasing concentration of 4-PBA (1–20 μM) which show two absorption peaks (Fig 2A). The major absorption peak is in the region of 200–230 nm attributed to n-π* transition of the protein backbone and 278 nm peak is due to microenvironment changes of tryptophan (Fig 2A, insets). It can be seen that adding increasing concentrations of 4-PBA causes a blue shift in the 280 nm region (Tryptophan absorption region) owing to changes in tryptophan microenvironment upon complex formation (Fig 2A; inset). There is a slight increase in the absorption peak of HSA due to its interaction with 4-PBA, which inflicts changes in the protein backbone, and thereby confirming complex formation between 4-PBA and HSA.

#### Fluorescence spectroscopy

Fluorescence emission spectroscopy showed an increase in emission of HSA with the gradual addition of 4-PBA (1–20 μM) (Fig 2B). Similar observations have been obtained upon binding of guaiacol [20] and betulinic acid [21] to HSA. The increase in the tryptophan fluorescence could be attributed to the shift of tryptophan towards a less polar microenvironment due to complex formation of 4-PBA with HSA.

The binding constant from the fluorescence experiments was determined by using the following equation [22]:

$$\frac{1}{\Delta F}=\frac{1}{{\Delta F}_{\max}}+\frac{\left(\frac{1}{K[L]}\right)}{\frac{1}{{\Delta F}_{\max}}}$$

where ΔF = F_x_-F_0_ and ΔF_max_ = F_∞_-F_0_; F_0_, F_x_ and F_∞_ are emission fluorescence intensities of free HSA, intermediate and saturation concentration of 4-PBA, respectively; K is the binding constant and [L] is the ligand concentration. Physiological ligands like fatty acids have K_d_ in the range of 10^4^−10^8^ M^-1^ [23], due to their ability in binding to multiple sites. 4-PBA interacts with a binding constant of 2.69 x 10^5^ M^-1^ (Fig 2D). Drugs and small molecules like flavonoids which prefer binding to Sudlow Site I or II have binding constant in the range of 10^4^−10^6^ M^-1^ [24]. This is governed by a variation in the type of binding assisted by polar or non-polar interactions mostly within the van der Waal interacting region of W214 (i.e. ~4.0Å).

The effect of ethanol alone on the fluorescence spectra of HSA in Tris HCl-NaCl at pH 7.4 was evaluated with an excitation at 295 nm. It can be seen that ethanol (used in diluted amounts) did not cause any alterations to the HSA conformation (Fig 2B).

#### 4-PBA does not displace Quercetin from Sudlow Site I at Subdomain IIA

**Fig 3:**
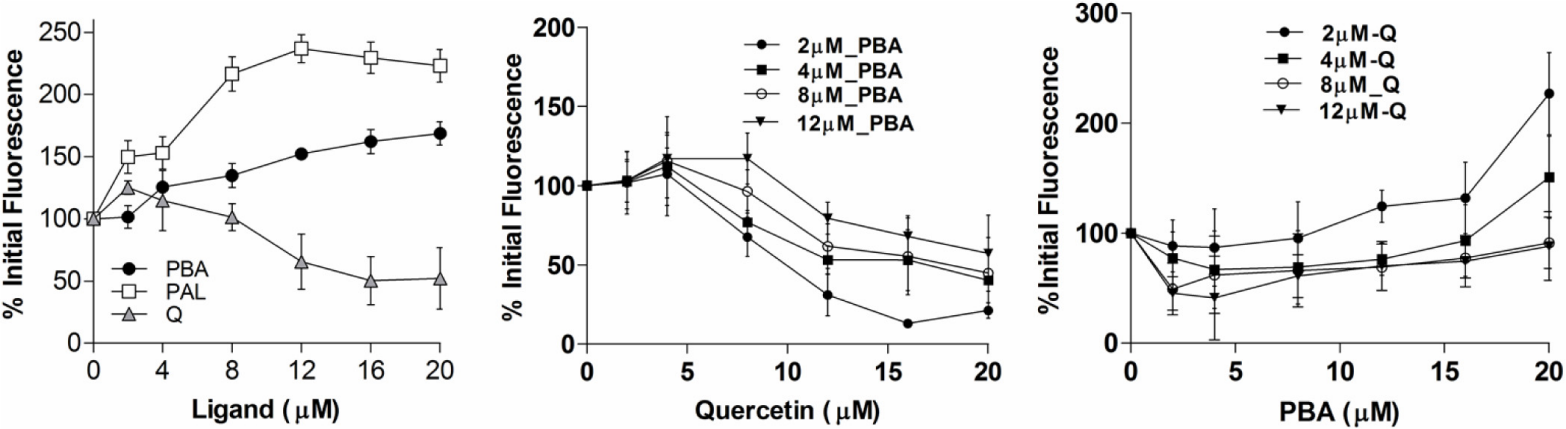
Ligand displacement assay of HSA with Quercetin, 4PBA. a) Percentage initial fluorescence of HSA (at 345 nm) upon addition of 4PBA, Palmitic acid and Quercetin (2–20 μM). b) Binding of 4PBA and Quercetin at different sites. Tryptophan fluorescence of HSA was monitored at 345 nm in the presence of 4PBA. To HSA-PBA complex (of varying PBA concentration: 2–12 μM), Quercetin was added from 2–20 μM and c) To HSA-Quercetin complex (of varying Quercetin concentration: 2–12 μM), PBA was added from 2–12 μM. HSA fluorescence was normalized to 100% in the absence of added ligands.

Increased emission fluorescence of HSA upon 4-PBA binding indicates increasing hydrophobic atmosphere of the lone tryptophan residue of HSA (Fig 3A). Ligands occupying Sudlow Site I quench tryptophan fluorescence as repeatedly observed in previous studies [25–27]. This indicated the possibility of 4-PBA occupancy at other sites like the abundant fatty acid binding sites (FA sites). To verify this selectivity, ligand displacement assay was performed with Dansylglycine which selectively binds to Sudlow Site II and Quercetin that selectively binds to Sudlow Site I. In fixed fluorescence measurements, decrease in the percentage fluorescence was observed when incremental concentration of Site I specific ligand Quercetin [28, 29] (2–20 μM) was added to HSA-4-PBA complex (Fig 3B).This clearly suggests that in the presence of 4-PBA, Quercetin specificity towards its binding at Sudlow Site I remains unaltered. In the reverse experiment when HSA-Quercetin complex was titrated (Quercetin concentration: 2–12 μM) with increasing concentration of 4-PBA (2–20 μM), a gradual increase in fluorescence was observed. This confirms the binding of 4-PBA to sites other than Sudlow Site I. Note that the observed reduction in percentage initial fluorescence when incubated with Quercetin is due to its quenching effect on tryptophan.

#### Displacement of Dansylglycine by 4-PBA

**Fig 4:**
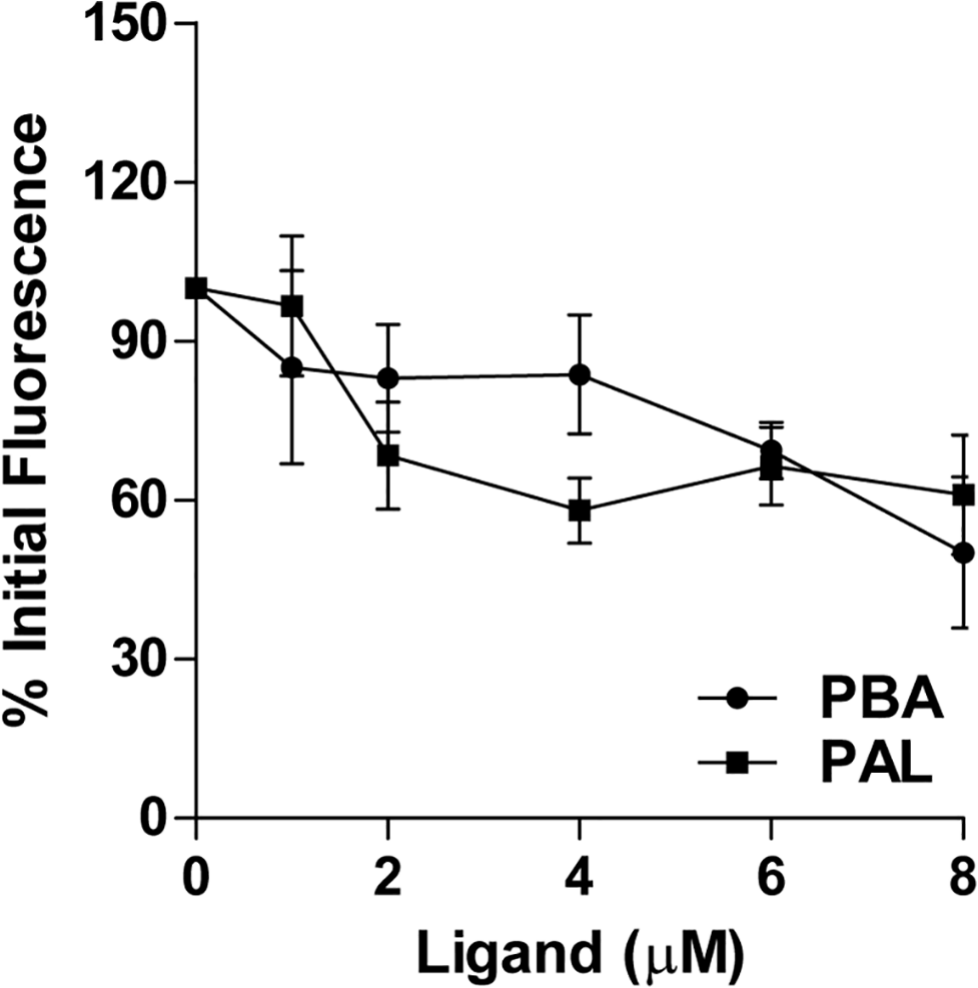
Displacement of Dansylglycine by 4PBA and Palmitic acid. 4PBA and Palmitic acid displacement of Dansylglycine. To HSA (1 μM) and Dansylglycine (1 μM) complex, 4PBA and Palmitic acid were added incrementally from 1–8 μM.

Non occupancy of 4-PBA at the Sudlow Site I and the increase in fluorescence of the 4-PBA-HSA complex suggested its occupancy at fatty acid binding sites or Sudlow Site II in HSA. To answer this, we explored the displacement of Dansylglycine [30]; a Sudlow Site II specific probe by 4-PBA and Palmitic acid. Fixed wavelength fluorescence emission for Dansylglycine has been recorded using 335 nm as excitation and 490nm as emission maximum. Results show significant decrease in dansylglcycine fluorescence (in HSA-Dansylglycine complex)with increasing concentrations of 4-PBA and Palmitic acid (Fig 4). The loss of fluorescence increased significantly at higher concentrations of 4-PBA (HSA:4-PBA molar ratio > 1:4) depicting displacement of Dansylglycine from Sudlow Site II. A similar displacement profile is also exhibited by Palmitic acid.

#### Binding and stabilization of 4-PBA at FA sites

The fluorescence experiments convinced us of the FA site binding possibility of 4-PBA. To further understand the binding specificity and structural changes that 4-PBA inflicts on HSA, molecular dynamics simulation of 4-PBA bound at six fatty acid binding sites was performed. The FA site (FA site 7) at subdomain IIA was omitted as 4-PBA did not show any quenching of tryptophan in the fluorescence experiments.

**Fig 5:**
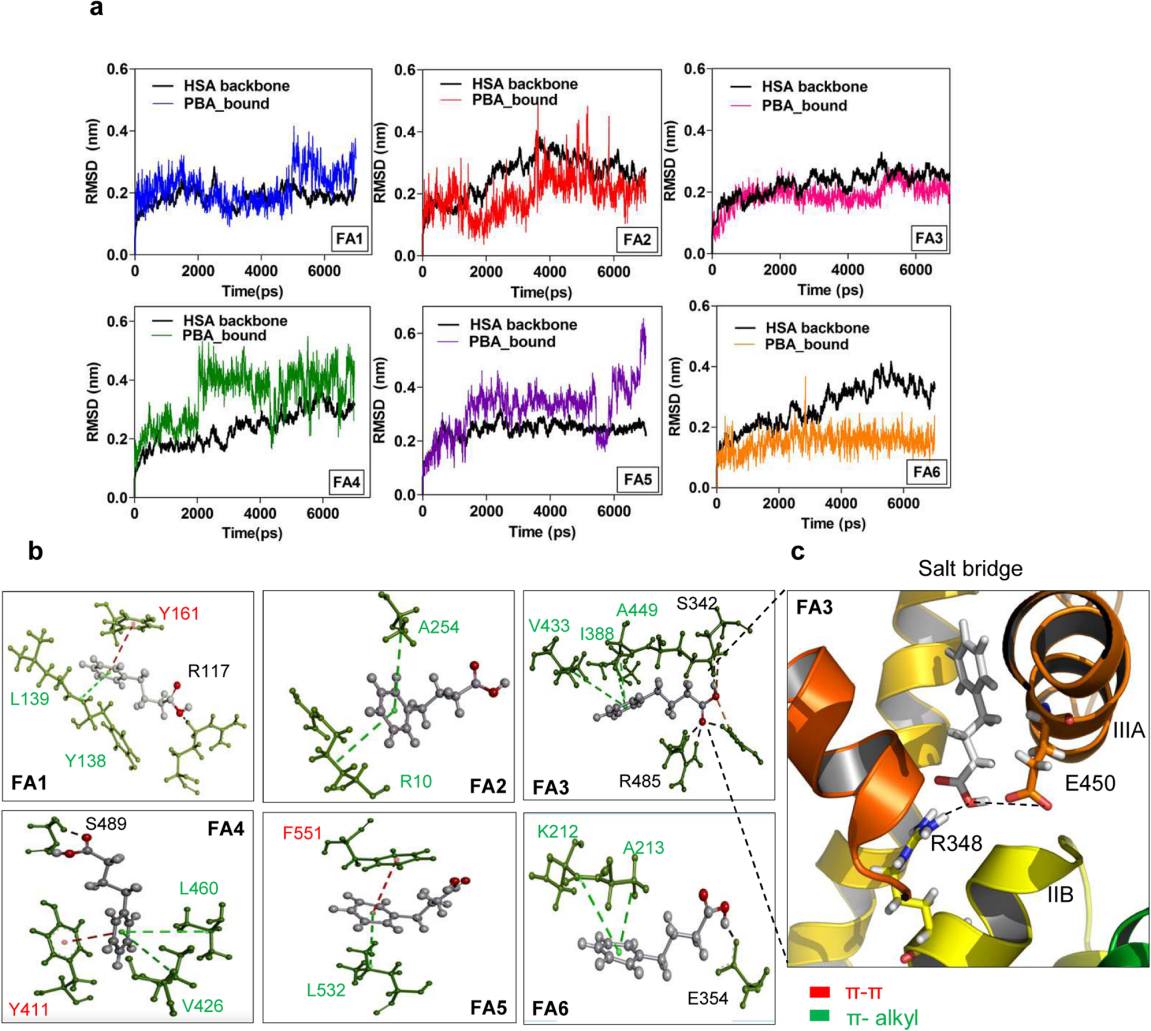
Stability of 4PBA at FA binding sites of HSA. a) Time evolution of RMSD of the HSA backbone and PBA bound forms during 7ns MD simulation of 4PBA bound to HSA at different FA binding sites. b) Interaction profile of 4PBA at all FA binding sites c) Salt bridge formation at FA3.

The molecular dynamics study displayed retention of 4-PBA at FA binding sites after completion of the simulation run. The RMSD’s along the trajectories were below 0.5 nm for all 4-PBA bound complexes but some significant differences were observed between the complex stabilization at high (FA Site 2, 4 and 5) and low affinity binding sites (FA Site 1, 3 and 6) (Fig 5A). Most of the backbone and PBA bound RMSD fluctuations can be attributed to the nature of the binding environment of 4-PBA at FA sites and the likely impact on the global fluctuation of various subdomains. 4-PBA binding at FA site 3 and 6 shows considerable stability of the RMSD due to H-bonding mediated by terminal OH of the 4-PBA–COOH group (Fig 5B). 4-PBA at FA6 complex RMSD shows stabilization throughout the simulation, due to stable H-bonding with E354 of Subdomain IIB (which shows the least fluctuation during the course of simulation). However, FA6 complex backbone remains destabilized because of the outward motion of the protein during the simulation run. Among the others, both backbone and PBA bound RMSD at FA3 site attains stability at about 1800 ps and shows better stabilization as compared to the starting structure. This stabilization is largely due to a bulk of H-bonded (Fig 5B) as well as non-covalent interactions (salt bridge) mediated by 4-PBA at FA3 site. As the FA3 site is present in close proximity to the Subdomain IIIB (most flexible domain in HSA), binding of 4-PBA limits the flexible motion of this domain thereby contributing greatly to its backbone stabilization. Much of the 4-PBA mediated internal motions of HSA occur at the Subdomain IIIB and IA (which encloses FA sites 5 and 2), majorly contributed to the RMSD fluctuations at both these sites in the entire simulation run. As the high-affinity fatty acid binding sites (FA sites 2, 4 and 5) have large hydrophobic cavities, 4-PBA with very short hydrophobic tail is preferably positioned at the origin of the cavity. Unlike long chain fatty acids, it shows lesser number of stabilizing hydrophobic interactions at these pockets that contribute majorly to the local fluctuation in FA sites 2, 4 and 5. Contrary to expectations, FA site 5 backbone RMSD showed good stabilization throughout the simulation. Typically extra stabilization of 4PBA-HSA complex RMSD profiles (observed at FA1, FA3 and FA6) is attributed to π-π, π-alkyl and H-bond interaction forces. Overall, 4-PBA binding at the low affinity FA binding sites showed better complex and backbone RMSD stabilization throughout the time course of the simulation.

In FA site 1, 3 and 6 we observe a stabilization of RMSD majorly contributed by stacked π-π, π-alkyl and H-bond. The other sites, i.e. FA site 2, 4 and 5 we observe some slight fluctuation in the RMSD profile due to no/limited contribution from H-bond interaction. To sum up the findings 4PBA prefers interacting majorly by stacked π-π (hydrophobic) and H-bond interaction (hydrophilic) in the low affinity FA binding sites and only by partially stacked π-π and π-alkyl interactions in the high affinity binding sites (S1 Table).

### Binding of 4-PBA to fatty acid binding sites induces protein conformational changes

#### Effect of binding of 4-PBA on the ellipticity changes in HAS

**Fig 6:**
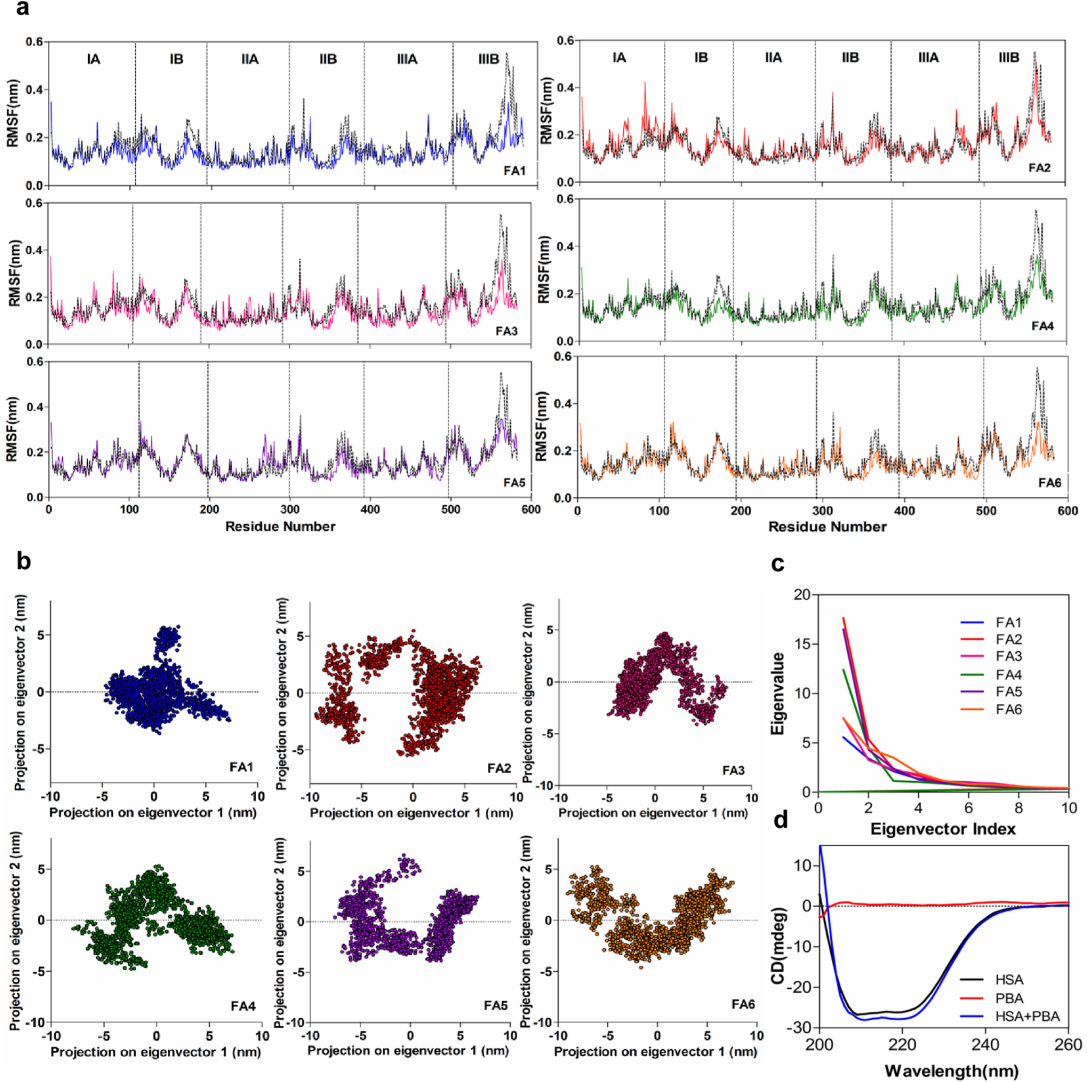
Binding of 4PBA induces conformational changes on HSA. a) RMSF of Cα atoms of Unliganded HSA (discontinuous black lines) and 4PBA bound HSA at different FA binding sites. The demarcations show different Subdomains of HSA. Significant fluctuations can be seen at Subdomain IA and IIIB; the most mobile and hydrophobic fragments of HSA. b) 2D projection of first two principal components of different 4PBA-HSA bound models. c) Spectrum of Eigenvalues vs Eigenvector Index. d) CD absorption spectra of HSA-4PBA complex (HSA-1 μM; 4PBA-1μM).

In order to analyse the effect of HSA-4-PBA complex formation on the changes in secondary structure of HSA, the CD spectra of HSA in the presence and absence of 4-PBA was obtained. The changes in HSA ellipticity is calculated using: MRE = θ/10rlC; where, MRE is mean residue ellipticity, θ is observed ellipticity expressed in millidegree, r = 585 (number of amino acid residues of HSA), l is the path length (0.1cm) and C is the molar concentration of HSA. Circular Dichroism analysis shows two major negative intensities at 208 and 222nm, characteristic of HSA alpha helix which shows significant decrease in negative ellipticity upon addition of 4-PBA (Fig 6D). This causes a slight increase in alpha helix percentage (38.8% in native HSA to 47.10% in HSA+4PBA) suggesting an increasing rigidity (stabilization) of the protein upon 4-PBA binding (S2 Table). However, the CD spectra of HSA shows similar shape for both in presence and absence of 4-PBA indicating predominant α helical content. This behaviour indicates overall movement and stabilization of HSA on binding to 4-PBA. This is further explored by molecular dynamics of HSA in an unliganded and different 4-PBA bound states.

#### Root Mean Square Fluctuations

The Root Mean Square Fluctuation of the Cα backbone was computed for the Unliganded HSA and HSA-4-PBA complex from b-factors (RMSF = √3B/(8π^2^)), where B is the b-factor. The RMSF profiles of HSA show high fluctuations in the C-terminal domain (IIIB) as compared to the N-terminal (IA) (Fig 6A); this is in agreement with earlier studies [31]. Much of the HSA fluctuations are observed in the Sub domain IA and IIIB of all FA binding sites (Fig 6A). Binding of 4-PBA stabilizes the internal motion of these two domains and contributes to increased stability. 4-PBA occupancy at nearly all FA sites stabilizes Subdomain IIA and IIB slightly (Fig 6A). Compared to these two subdomains, other subdomains are mostly rigid and do not confer much to the 4-PBA induced conformational changes.

#### Principal Component Analysis

**Fig 7:**
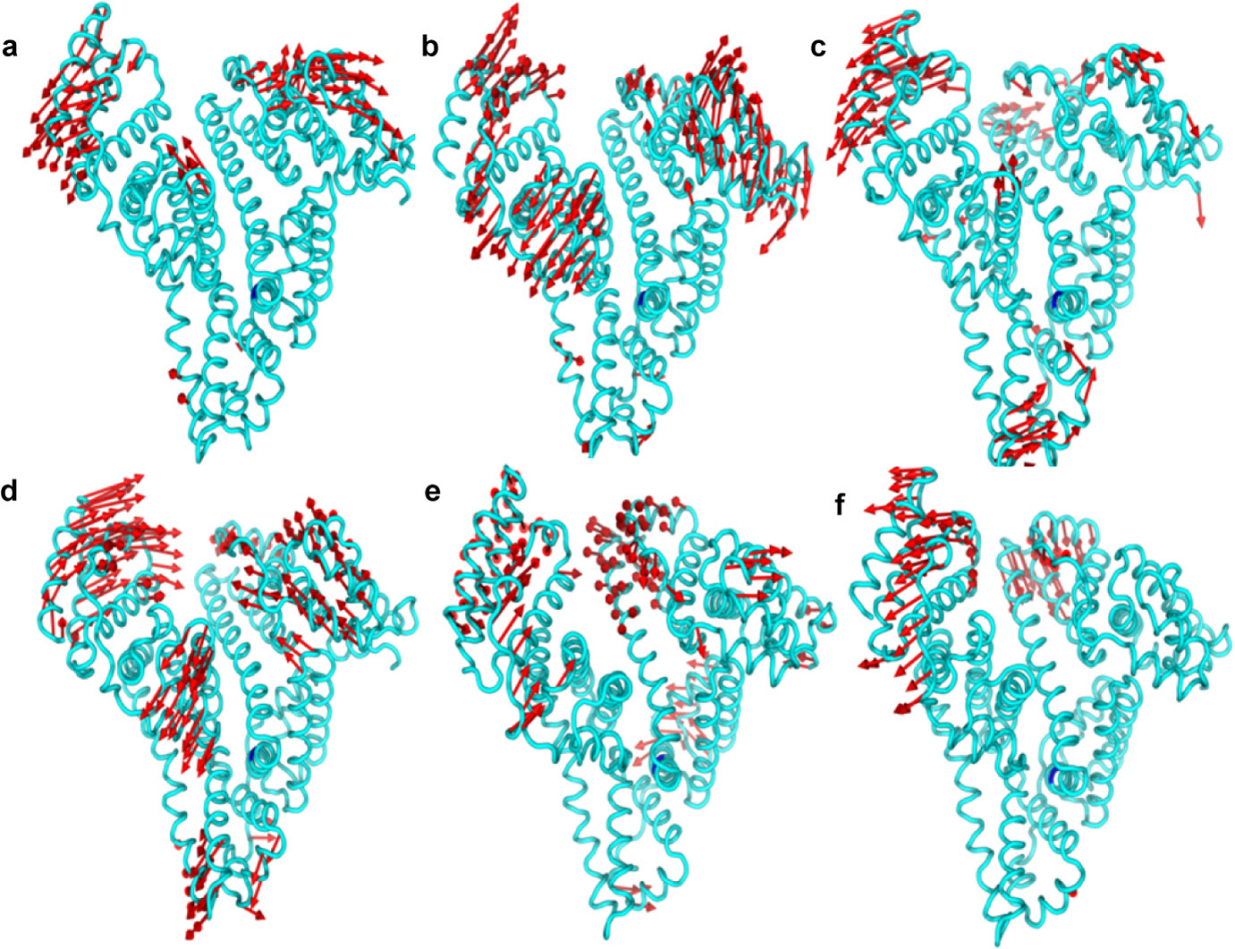
Motion of Cα atoms for the extreme values of the principal components obtained from MD simulation trajectory. a-f) represents the motion of Cα atoms of FA site 1 to 6- 4PBA occupied complex respectively.

The simulation trajectory of 4-PBA-HSA complexes were analysed as two principal components and the calculated eigenvectors were assembled in order of decreasing eigenvalues. The relative contribution to protein fluctuation was obtained from the first 10 modes which was responsible for more than 90% of the protein motion. PCA analysis (Fig 6B) showed that 4-PBA occupancy at Sudlow Site II (FA3 and FA4; subdomain IIIA) and low affinity binding sites (FA1 and FA6) occupied less conformational space as compared to 4-PBA bound to high affinity binding sites like FA2 and FA5. Among the top ten eigen values, the first two accounted largely for protein motion in each complex (Fig 6C). Long chain fatty acids binds linearly to the FA2 and FA5 sites (due to their large binding cavities), and nonlinearly at FA 1, 3 and 6 due to internal strain (owing to smaller cavity space) [31]. Whereas, the 4-PBA carbon chain being very small, facilitates stable interaction at much smaller cavities localized at FA1, 3, 4 and 6 causing less protein motion. This strained conformational space residence further confirms the RMSD and RMSF data which shows comparable protein motions due to localized stabilization owing to H-bonding at FA sites 3, 4, and 6 (Fig 5A and 5B). Almost similar Subdomain IIIB motional drift is observed for 4-PBA bound complexes at FA1, 3 and 6 which is contrary to that observed in FA2, 4 and 5 occupied complexes (Fig 7). 4-PBA engaged at FA2, 4 and 5 shows much flexing motion (of two most mobile domains; Subdomains IA/B and IIIB) as compared to the opening motion when bound to FA sites 1, 3 and 6. All these binding modes i.e. occupancy of FA sites 1,3,4 and 6 induced relative conformational drifts of Subdomain IIIB and thereby contribute to the dynamic motion of W214.

#### MM/PBSA for binding of 4-PBA at fatty acid binding sites on HSA

In order to decipher the selectivity and explain the difference in binding patterns of Palmitic acid and 4-PBA, the binding free energy of 4-PBA bound at different FA binding sites was computed. Table 1 shows the free energy components of various 4-PBA-HSA complexes. The highest negative binding free energy can be observed for 4-PBA-HSA complex at FA3 followed by FA 6,1,5,4 and 2 (i.e. 3>6>1>5>4>2). Although 4-PBA-HSA complex at FA4 is much more stable than FA5, the increase in binding free energy observed at FA5 site is due to the compensation by electrostatic interactions. This binding nature further demonstrates that 4-PBA binding is mostly governed by the possibility of stable contacts observed at the opening of the cavity when it binds at FA4 and 6. This is unlike long chain non-esterified fatty acids like Palmitic acid, which linearly incorporates at larger hydrophobic cavities.

**Table 1:** MM-PBSA Binding Free Energy components of HSA-4PBA complex.

Binding site	∆E _*coul*_ *	∆E _*LJ*_ *	∆G _*polar*_	∆G _*nonpolar*_	∆G _*binding*_
**FA1**	-63.417 ± 3.536	-114.161 ± 2.364	102.061 ± 3.514	-11.68 ± 0.142	-87.197 ± 3.481
**FA2**	-21.932 ± 2.326	-112.137 ± 1.843	84.129 ± 4.651	-12.088 ± 0.061	-62.028 ± 4.902
**FA3**	-226.546 ± 3.722	-102.734 ± 2.204	202.788 ± 3.920	-11.414 ± 0.052	-137.906 ± 4.208
**FA4**	-8.085 ± 3.780	-110.249 ± 1.194	54.044 ± 2.653	-11.976 ± 0.122	-76.266 ± 2.079
**FA5**	-60.194 ± 10.552	-109.691 ± 2.423	100.723 ± 8.327	-11.88 ± 0.090	-81.042 ± 3.809
**FA6**	-99.504 ± 3.568	-113.63 ± 1.632	112.627 ± 3.415	-11.647 ± 0.046	-112.153 ± 2.845

All energy values are expressed in KJM^-1^.

*LJ = Lennard Jones Potential

*Coul = Coulombic Charge

∆G _*binding*_ was calculated from Eq 1

Binding free energies as computed by the MMPBSA method varied from the spectroscopic binding free energy. Similar discrepancies have been observed by Fujiwara et al [19] between experimental and theoretical calculations. Perhaps, computationally expensive method like normal mode analysis with very high simulation time >50ns will allow much conformational freedom of HSA to minimize this discrepancy.

#### Dynamic Movement of W214 and changes in hydrophobicity on 4-PBA occupancy at different FA sites

As W214 fluorescence shifts are affected by changes in its polar microenvironment, the next step is to compare this with molecular dynamics studies. Movement and hydrophobicity changes of W214 and W214 pocket descriptor residues in (a) unliganded HSA and (b) HSA occupied by 4-PBA at individual FA binding sites by SASA analysis is compared (Table 2). The results show dynamic movement of W214 upon 4-PBA binding. The major contribution to W214 hydrophobicity is derived from its internal positional shift or movement of important descriptor residues from Subdomain IIA, IIB and IIIA which forms the W214 pocket. Because these movements alter the solvent accessibility of W214, when 4-PBA occupies various FA binding sites, the independent or co-ordinated motions of these Subdomains recompenses the W214 hydrophobicity.

**Table 2:** Total and Residue Hydrophobicity (SASA analysis).

Binding site	Hydrophobicity of W214*	Total Hydrophobicity HSA-4PBA*
**Unliganded HSA**	0.77±0.122	136.556±0.25
**FA1**	0.74±0.125	139.159±0.220
**FA2**	0.81±0.111	138.709±0.220
**FA3**	0.72±0.114	140.541±0.212
**FA4**	0.91±0.140	144.522±0.215
**FA5**	0.91±0.147	139.153±0.226
**FA6**	0.67±0.116	143.883±0.214

*Values are expressed in nm^2^

**Fig 8:**
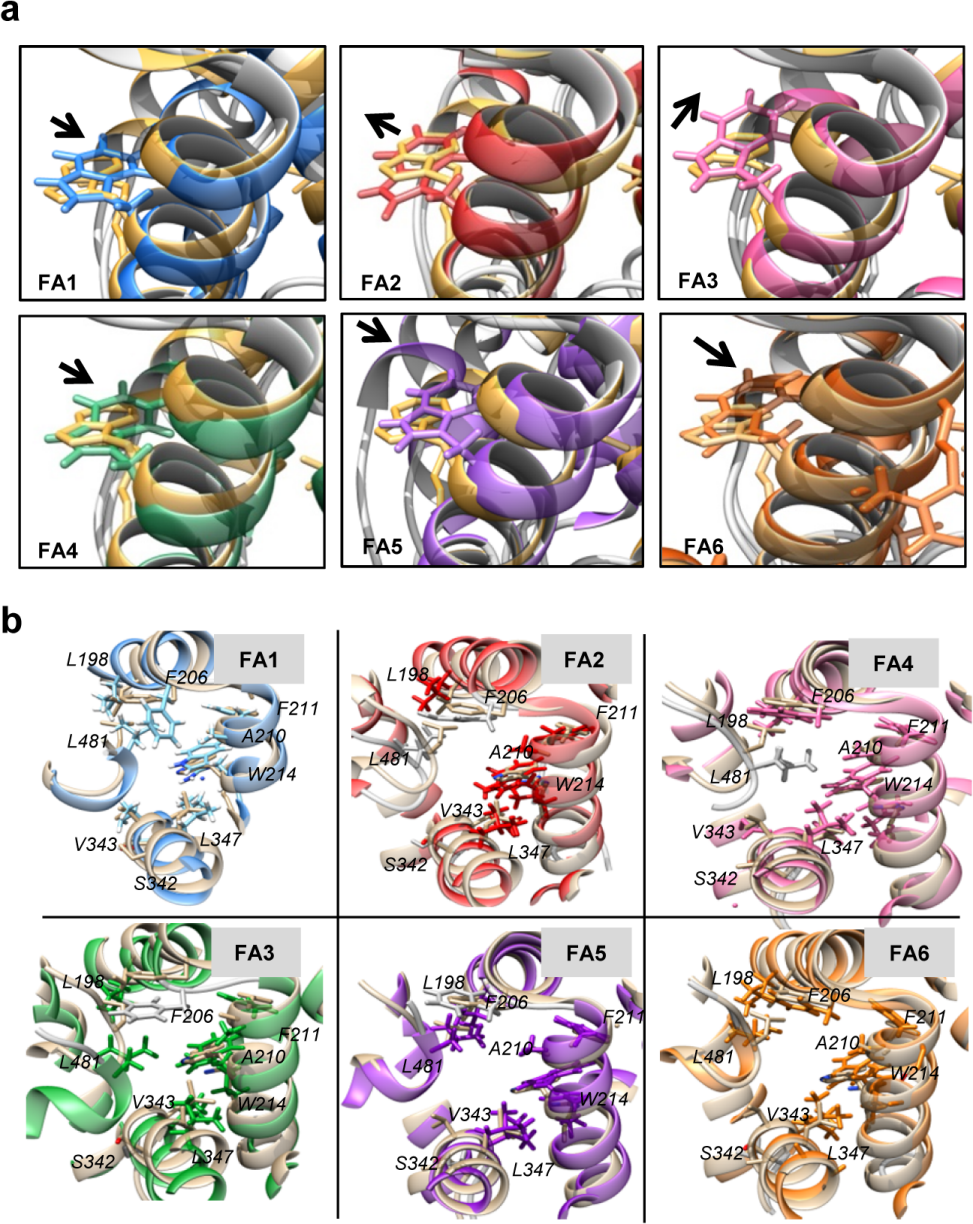
Dynamic Movement of W214 and descriptors of W214 pocket at HSA-4PBA complexes. a) Shifting of W214 residue in different HSA-4PBA complex. b) Movement of W214 pocket descriptors upon 4PBA binding at different FA binding sites.

Table 2 shows W214 hydrophobicity and total hydrophobicity changes of HSA upon 4-PBA occupancy at FA binding sites. Irrespective of its occupancy at any FA site, and difference in the W214 hydrophobic SASA observed in each of these complexes, an overall increase in total hydrophobicity of HSA upon binding of 4-PBA is observed. This can be understood from the movements of W214 and important residues of its W214 binding pocket (Fig 8). The net increase in hydrophobicity of the pocket is compensated either by the internal movement of W214 or by important residues like L198, F206, A210, F211 (Subdomain IIA), V343, L347 (Subdomain IIB) and L481 (Subdomain IIIA) (Fig 8B; Table 3). The residue W214 moves outwards when 4-PBA complexes with HSA at FA 1, 4, 5 and 6 but inwardly at FA 2 and 3 (Fig 8A). This discrepancy in the internal movement and hydrophobicity is resolved by the movement of the descriptor residues of the Subdomain IIA, IIB and IIIA.

**Table 3:** Residue hydrophobicity of descriptors of W214 pocket upon different FA site complexes (SASA analysis).

Binding Site	Descriptors of W214 binding pocket (values are expressed in nm^2^)						
	L481	L198	F206	A210	F211	V343	L347
**Unliganded HSA**	0.79±0.14	0.47±0.09	0.86±0.21	0.51±0.09	0.20±0.07	0.14±0.06	0.43±0.09
**FA1**	0.79±0.11	0.55±0.11	0.95±0.17	0.51±0.07	0.25±0.08	0.17±0.06	0.37±0.08
**FA2**	0.71±0.14	0.38±0.09	0.87±0.14	0.51±0.09	0.24±0.21	0.14±0.05	0.35±0.07
**FA3**	0.82±0.13	0.43±0.07	1.0±0.16	0.48±0.08	0.19±0.08	0.22±0.06	0.38±0.09
**FA4**	0.69±0.11	0.57±0.08	0.85±0.11	0.45±0.08	0.24±0.14	0.30±0.08	0.27±0.07
**FA5**	0.63±0.09	0.41±0.08	0.71±0.14	0.46±0.12	0.17±0.08	0.19±0.06	0.33±0.06
**FA6**	0.66±0.16	0.60±0.11	0.76±0.17	0.36±0.10	0.26±0.07	0.23±0.07	0.29±0.09

So, an overall increase in hydrophobicity leads to the stabilization of HSA bound 4-PBA as compared to unliganded HSA. The increased W214 hydrophobicity explains the rise in fluorescence emission intensity when HSA is titrated with increasing concentrations of 4-PBA.

## Discussion

Human serum albumin is the most important multidrug transporter in our body and drug binding to HSA is of important pharmacokinetic relevance. Binding nature of chemical chaperone 4-PBA to HSA is investigated by a combination of approaches. Absorption spectroscopy shows a stable complex formation between HSA and 4-PBA (Fig 2A), with a mild alteration to the protein backbone corroborated further by CD analysis. The absorption spectra showed gradual broadening and a small blue shift in the tryptophan absorption region upon gradual addition of 4-PBA, confirming alterations in the molecular environment of tryptophan. Spectrofluorimetry assays show an increase in the tryptophan emission spectra on gradual addition in molar concentration of 4-PBA (Fig 2B). This is concurrent with the slight increase in the quantum yield or decrease in the polarity of tryptophan. As tryptophan emission is highly susceptible to changes in polarity of its ambient environment [32], this data suggests the possibility of tryptophan exposure to more hydrophobic environment.

Like non-esterified fatty acids, 4-PBA can bind to most of the major fatty acid binding sites non-covalently, but its principal binding sites vary. Fluorescence assays show an increase in relative percentage fluorescence of HSA when titrated with increasing concentrations of Palmitic acid and 4-PBA (Fig 3A). Palmitic acid shows at least a fold increase in percentage fluorescence when compared with 4-PBA. Palmitic acid stabilizes the hydrophobic pockets at Subdomain IIIB and IA (where high affinity FA sites 5 and 2 are present) to a much greater extent when compared to 4-PBA. In the ligand displacement assay, prior addition of Quercetin to HSA did not prevent the increase in the fluorescence upon adding 4-PBA (Fig 3C). On the contrary when 4-PBA was pre-incubated with HSA, addition of Quercetin further quenched fluorescence (Fig 3B). This clearly shows that 4-PBA has preferential binding to sites away from Sudlow Site I. (Fig 3B). Subdomain IIA binding of 4-PBA is excluded as it does not show W214 fluorescence quenching. 4-PBA binding at FA site 3 shows the highest negative binding free energy as shown by MMPBSA (Table 1) and least conformation space in its PCA analysis (Fig 6B). These findings are in concert and point out that 4-PBA prefers binding at Subdomain IIIA at FA sites 3 and 4 (Sudlow Site II). This is further supported by ligand displacement studies using Site II probe Dansylglycine (Fig 4). 4-PBA at molar excess can displace dansylglycine from Site II showing its binding efficacy at this site.

Upon binding, 4-PBA stabilizes the overall conformation of HSA. This behaviour is similar to that of long chain non-esterified fatty acids like Palmitic acid and Myristic acid [32], [33]. From earlier studies it is well known that binding of fatty acids to HSA produces dramatic conformational changes [34]. Domains I and III get displaced to the left thus opening up the central crevice. A similar behaviour is observed for 4-PBA binding (Fig 7) and molecular dynamics simulation with a partially and completely bound model of HSA-4-PBA complex could reveal a better picture. FA2 site is at the interface of domain I and II and long chain fatty acid binding at this site stabilizes the movement of domain I. As 4-PBA binding takes place at the origin of this cavity it is unable to inflict similar stabilization.

In the MD simulation of 4-PBA bound to HSA at six FA binding sites, 4-PBA forms stable H-bonding at FA site 3, 4 and 6 (Fig 5B). In FA4 site, it forms stable hydrogen bonding with S489 at a distance of 2.8 Å. It forms a π-π stacking interaction with Y411 at 3.52 Å (Fig 5C). This binding is similar to the binding behaviour of nonharmane [35] and Bcl2 family anticancer agent [36] as previously reported. In addition to this, the FA4 backbone stabilization role of π-alkyl interactions with Subdomain IIIA residues V426 at 4.91Å and L460 at 5.17 Å is observed. When considering 4-PBA binding at FA3, an overall stable complex formation is seen from the RMSD, RMSF and PCA. This particular stability can be attributed to linear occupation of 4-PBA and formation of three stable hydrogen bonds with R485, E450 and R348. A salt bridge created between the domain III and II via supplanting of E450 (Subdomain IIIA) by 4-PBA to R348 (Subdomain IIB) contributes greatly to the stabilization of the mobile subdomain IIIB. As a result of this, a very stable RMSD is observed during the course of simulation when 4-PBA occupies FA3 site. Apart from this it is also stabilized by a large number of Coulombic interactions (Table 1). Moreover the high selectivity of 4-PBA to bind towards FA3 site (subdomain IIIA) can allow cooperative binding of physiological ligands like FA’s. In pathological states like obesity, where there is an increase in circulating free fatty acids [37], 4-PBA binding to HSA might be affected. Having obtained some hints on this phenomenon, it would be interesting to find out the pharmacokinetics of 4-PBA in obese patients in clinical dosages.

## Conclusions

This work shows the binding nature of chemical chaperone 4-PBA with human serum albumin. A cumulative analyses with different methods show that 4-PBA can bind to FA binding sites in HSA with high specificity of binding at FA site 3 (subdomain IIIA). Like physiological FA’s it can inflict similar conformational changes upon interaction to HSA. Transport of 4-PBA will largely vary due to increased concentrations of FA’s in the plasma in pathological states like obesity. This study gives better perspective on future pharmacokinetic studies of 4-PBA and so clinically relevant changes in dosage can be profiled for varying disease states.

## Supporting Information

S1 Table Interaction forces of 4PBA at different FA binding sites of HSA. (DOCX)

S2 Table Secondary structure evaluation from far UV CD spectra. (DOCX)
