# Supplementary material for: Evidence that Chemical Chaperone 4-Phenylbutyric Acid Binds to Human Serum Albumin at Fatty Acid Binding Sites
Source: PLoS One. 2015 Jul 16;10(7):e0133012. doi: 10.1371/journal.pone.0133012 (PMC4504500; doi:10.1371/journal.pone.0133012)
Supplement: S1 Table — (DOCX) [file pone.0133012.s001.docx]

**Supporting Information**

**S1 Table. Interaction forces of 4PBA at different FA binding sites of HSA**

| **FA Sites** | **Interacting Residues of HSA** | | |
| --- | --- | --- | --- |
|  | **π-π** | **π-alkyl** | **H-bond** |
| FA1 | Y161 | Y138 | R117 |
| FA2 |  | A254, R10 |  |
| FA3 |  | V433,A449,I388 | S342, R485 |
| FA4 | Y411 | L460, V426 | S489 |
| FA5 | F551 | L532 |  |
| FA6 |  | K212, A213 | E354 |
