# Supplementary material for: Evidence that Chemical Chaperone 4-Phenylbutyric Acid Binds to Human Serum Albumin at Fatty Acid Binding Sites
Source: PLoS One. 2015 Jul 16;10(7):e0133012. doi: 10.1371/journal.pone.0133012 (PMC4504500; doi:10.1371/journal.pone.0133012)
Supplement: S2 Table — (DOCX) [file pone.0133012.s002.docx]

**Supporting information**

**S2 Table. Secondary structure evaluation from far UV CD spectra**

| **Secondary Structure** | **HSA (%)** | **HSA+4PBA (%)** |
| --- | --- | --- |
| Helix | 38.80 | 47.10 |
| Beta | 14.50 | 0.50 |
| Turn | 20.40 | 26.60 |
| Random | 26.20 | 25.80 |
